# A qualitative study of family members’ perspectives regarding decision-making for nursing home residents’ care

**DOI:** 10.1080/17482631.2024.2370545

**Published:** 2024-06-21

**Authors:** Anne Helene Mortensen, Dagfinn Nåden, Dag Karterud, Ann Gallagher, Vibeke Lohne

**Affiliations:** a Faculty of Health Sciences, Department of Nursing and Health Promotion, OsloMet, Oslo, Norway; b Department of Health Sciences, Brunel University London, London, UK

**Keywords:** Qualitative research, hermeneutical interpretation, interview, nursing homes, family, dignity, paternalism, autonomy, refusal of care, decision making

## Abstract

**Purpose:**

We explored how family caregivers perceive decision-making regarding the care of nursing home residents.

**Methods:**

This qualitative study used Flemming’s Gadamerian-based research method. In person semi-structured interviews about decision-making concerning residents’ care were conducted with 13 family members (nine women, four men) of residents of three Norwegian nursing homes.

**Findings:**

The following themes emerged: Excessive focus on autonomy threatens resident wellbeing and safety. Resident wellbeing is the caregiver’s responsibility. Resident wellbeing serves as a guiding principle.

**Conclusions:**

The family members of residents and the nursing home caregivers disagreed about the significance of upholding resident autonomy to respect residents’ dignity. The family members held that not all instances where residents refused care reflect autonomy situations as care refusal often does not reflect the resident's true values and standards but rather, stems from barriers that render necessary care actions difficult. In situations where residents refuse essential care or when the refusal does not align with the residents second-order values, the family members suggested that caregivers strive to understand the causes of refusal and seek non-coercive ways to navigate it. Hence, the family members seemed to endorse the use of soft paternalism in nursing homes to safeguard residents’ wellbeing and dignity.

## Introduction

Healthcare services were originally characterized by paternalism, with healthcare professionals having more power than patients and residents. Recently, focus has shifted towards strengthening resident autonomy in nursing homes (Moilanen, Kangasniemi, et al., 2021; Moilanen, Suhonen, et al., 2021). However, professional caregivers in nursing homes face ethical dilemmas when the principles of beneficence and autonomy conflict (Mortensen et al., 2022). The primary concern within discussions about autonomy and beneficence is paternalism (Jacobson & Silva, 2010; Killmister, 2018). Additionally, maintaining resident autonomy has been used as justification for not providing residents with necessary care (Moilanen, Suhonen, et al., 2021; Mortensen et al., 2022). This indicates the need for new research-based knowledge on the experiences of stakeholders regarding decision-making in nursing homes, especially regarding the use of paternalism. In a previous study, we explored how nursing home residents experience paternalism. In this study, we explore how family caregivers perceive decision-making regarding the care of nursing home residents.

## Background

The decision to move an individual to a care home is typically delayed until they can no longer manage with support from family and homecare services (Samsi et al., 2022). Hence, nursing home residents are older and frailer than before, and have a higher care dependency (Ekström et al., 2019; Rijnaard et al., 2016), which threatens resident autonomy (Caspari et al., 2018; Moilanen, Suhonen, et al., 2021). Supporting resident autonomy is perceived as a sign of respect and dignity, while hindering it leads to feelings of confinement and frustration (Moilanen, Kangasniemi, et al., 2021). Additionally, supporting independence and autonomy is crucial for residents’ adjustment when moving to a nursing home (Caspari et al., 2018; Davison et al., 2019). Nurses perceive autonomy as a basic principle and an important part of quality care. Caregivers in nursing homes support resident autonomy by protecting residents’ rights, acting as their advocates, and respecting their wishes. Nurses also support autonomy by offering choices and information to residents and their family members. Individualizing care and ensuring safety are also essential for supporting resident autonomy (Moilanen, Suhonen, et al., 2021). The belief that nursing home residents cannot make autonomous decisions and the need to balance residents’ wishes against those of their family members, sometimes hinders caregiver support of resident autonomy (Moilanen, Suhonen, et al., 2021). Family members and friends are important for supporting resident autonomy as they provide continuity to residents’ previous life experiences (van Loon et al., 2021).

Family members of nursing home residents are highly involved in making decisions regarding resident care (Egan et al., 2023; Mortensen et al., 2023), and are usually responsible for moving residents to the nursing home (Seiger Cronfalk et al., 2017). Family members often hold themselves responsible for monitoring and evaluating the quality of care the resident receives, and they have moral concerns and troubled consciences regarding the resident (Ekström et al., 2019; Seiger Cronfalk et al., 2017). Family members of nursing home residents have “insider knowledge” of resident history and can aid in the continuity of the resident’s values, beliefs, and personal identity, which is associated with adjustment, satisfaction, and healthy transition to the nursing home (Fitzpatrick & Tzouvara, 2019). The extensive knowledge and interest of family members contribute to their ability to detect subtle changes in residents and resident health that professional caregivers are unable to perceive (Powell et al., 2018). Family members also represent the resident as an intermediator between them and nursing home staff when they no longer can speak adequately for themselves (Ekström et al., 2019; Fitzpatrick & Tzouvara, 2019). The continuation of relationships with family members and significant others helps residents maintain self‐identity and helps bridge the past and the present (Fitzpatrick & Tzouvara, 2019; Seiger Cronfalk et al., 2017). Hence, the exchange of care-related information between family members and nursing home staff and family member participation in nursing homes is crucial for person-centred care (Ekström et al., 2019). However, in a review of 34 studies concerning facilitators and inhibitors to successful transition to long-time care facilities, only five studies investigated the perspective of family members (Fitzpatrick & Tzouvara, 2019), indicating the need for further research.

Existing studies have mostly explored the perspectives of nurses and residents regarding supporting resident autonomy and dignity in nursing homes (Moilanen, Kangasniemi, et al., 2021; Moilanen, Suhonen, et al., 2021), and research on the perspectives of family members, especially regarding paternalism, is lacking. The aim of this qualitative study is to explore how family caregivers perceive decision-making regarding the care of nursing home residents.

## Methodology

This study draws upon Gadamer’s philosophical hermeneutics, which asserts that our preconceived understanding of the world—our “horizon of understanding” – preconditions and shapes our interpretation of the world (Gadamer, 2013). This horizon is made up of all our knowledge, beliefs, biases, including unconscious and internalized understandings (Gadamer, 2013). Gadamer also emphasizes the importance of historical and cultural context in shaping our understanding (Gadamer, 2013). Thus, understanding is not an endpoint of interpretive research, but rather a precondition that evolves throughout the project.

In this study we used Flemming’s Gadamerian-based research method (Fleming et al., 2003). In accordance with this method our study proceeded in five stages: In the first stage, we established the research question in line with interpretative hermeneutics’ objective. In the second stage, we sought to comprehend and analyse our pre-existing beliefs about decision-making in nursing homes. Through discussions and reflections within the research group our biases and preconceptions were revealed. These preunderstandings indicated that the concepts of autonomy, paternalism, and dignity would contribute to enhance our understanding of the views of residents’ family members regarding decision-making. In the third stage of the study, the data collection, the first author engaged in dialogue with study participants through semi-structured interviews. In the fourth stage, our understanding was expanded through dialogue-based analysis and interpretation of the interviews, prior studies, and theoretical texts. The final stage of Flemming’s Gadamer-based research method involves ensuring the study’s trustworthiness by meticulously outlining each step of the research process to foster transparency and auditability.

### Theoretical framework

The interpretations of this study are influenced by our understanding of the concepts of autonomy, paternalism, and dignity.

The concept of autonomy refers to the right human beings have to self-government: the right to make own decisions, to decide own values and ends, and to pursue these. The concept of autonomy includes the ability to make autonomous decisions, which implies the capacity for rationality and critical reflection (Killmister, 2018). Additionally, the concept of autonomy describes conditions to determine if a choice is autonomous or not. For a choice to be autonomous, it must be motivated by second-order values and desires, must be justifiable by the one making the choice, and it cannot be a product of oppression or manipulation (Killmister, 2018). Second-order values and desires are those values and desires that we desire to have. These are long-held values and desires that align our actions with our long-term goals, as opposed to first-order values that align our actions to short-term goals and immediate desires (Killmister, 2018).

Disregarding a resident’s autonomy with the intention to protect or benefit the resident is considered paternalism. Hence, whenever autonomy and beneficence are discussed, paternalism is the primary concern (Jacobson & Silva, 2010). Acting against a person’s explicit wishes is usually considered hard paternalism (Killmister, 2018). If the choice is not truly autonomous or the resident’s capacity for autonomy is compromised, it is considered soft paternalism. Soft paternalism aims to bring a person’s choices and actions in line with their true goals and values. Hence, soft paternalism assumes a mismatch between expressed will and authentic will (Killmister, 2018). Soft and hard paternalism can be differentiated based on how easy the paternalistic influence is to resist. According to this conceptual framework, nudging is considered soft paternalism (Thaler & Sunstein, 2008), whereas pressure and coercion are considered hard paternalism.

Traditional definitions of autonomy emphasize independence, self-sufficiency, and the ability to think rationally. These virtues are also considered dignitarian norms, norms that carry dignity, which means that those who uphold them are bestowed with dignity (Killmister, 2020).

According to Killmister (2020), dignity consists of three intertwined strands: Status dignity, which asks others to treat us appropriate to the status or position we hold (human being, doctor, resident etc.), personal dignity, and social dignity. Personal and social dignity are subjective concepts that are linked to the adherence to applicable norms and standards. These are norms that the individual and/or society find dignifying to uphold, or view as disgraceful and degrading to violate. Thus, we have personal and social dignity to the extent to which we uphold or transgress the relevant norms. Transgression of these norms entails withheld esteem that can extend to disdain and shame (Killmister, 2020). Status dignity is objective and cannot be reduced but is harmed when others fail to treat us with the respect our status commands (Killmister, 2020).

### Study setting and participants

This study is part of a larger study exploring the experiences of different stakeholders in Norwegian nursing homes. The larger study includes participant observation across three different nursing homes and interviews with residents, staff, and family members. This article only reports findings from interviews with residents’ family members.

The participants were purposely sampled from a population of family members of residents at three different nursing homes: a long-term unit, a dementia unit, and a rehabilitation unit. The first author conducted the interviews between November 2020 and June 2021. The chosen nursing home units were diverse in resident population, caregivers’ experience with paternalism, and access to resources. Therefore, we assumed that the participating family members would also provide differing experiences and perspectives. Thirteen family members of residents, who were between 51 and 83 years of age, were interviewed. The number of included participants mirrored the sizes of the nursing home units.

The inclusion criterion was a family member of a resident in a participating unit who visited regularly. The sample was purposely recruited, with the aim of achieving a balanced gender distribution in both participating family members and residents (one family member per resident), variance in residents’ physical and mental function, and variance in the resident’s history of accepting and/or resisting healthcare and assistance. Registered nurses in the unit who had extensive knowledge of residents and their family members, owing to their involvement in care, recruited family members for participation. Participant selection for interviews was conducted at the end of a participant observation period (after five to six weeks), to ensure consensus between the researcher and the nurses concerning inclusion criteria and the need for a diverse sample of participants.

The characteristics of the residents and their participating family members are presented in Table 1.

**Table 1.** Sample characteristics.

Long-term unit Thirty residents	Informant	Relationship
Family members of two male residents and four female residents One resident was considered clear and oriented by their familyand nurse. Three residents had mild dementia, and two residentshad more serious dementia (according to the interviewed family members).	P1	Wife
	P2	Daughter
	P3	Daughter
	P4	Son
	P5	Son
	P6	Daughter
**Dementia unit** Eight residents		
Family members of one female and one male resident Both residents suffered from serious dementia and had a history of serious refusal of care.	P7	Daughter
	P8	Wife
**Short-term/rehabilitation unit** Twenty-seven residents		
Family members of two male residents and three female residents One resident had serious dementia and was awaiting transfer to a long-term care facility. One resident had mild dementia, according to their family member. One resident was mentally disabled, and two residents were considered clear and oriented by their family members.	P9	Daughter
	P10	Daughter
	P11	Son
	P12	Niece
	P13	Son

### Data collection

The semi-structured interviews were conducted in privacy in small meeting rooms in the nursing homes right before or after family visits. The interviews lasted between 30 and 65 min (mean: 46 min). Participants were assured that information provided in the interviews was confidential before the recordings began. Participants were aware of the researcher’s background in teaching and supervising nursing students and had been informed that the researcher was conducting participant observation in the nursing home. Data from the participant observation were not included in this study. However, observations did provide context during the interviews as they gave the researcher and participants a common frame of reference, which we believe enhanced the quality of the interviews. The interview guide contained open-ended questions encouraging participants to talk about what they considered important for the care of their resident, what contributed to successful collaboration between residents and staff, how nursing home staff could arrange for the best possible care for the resident, whether they had experienced situations where the resident had refused necessary care, and if they had experienced situations where coercion was used in the nursing home. The participants were also asked how staff should handle situations where residents resisted care activities such as food, morning routines, showering, and so on, how staff can prevent situations that may entail the use of coercion, and the extent to which staff can influence residents’ behaviours and choices before the influence becomes unethical. Silence, follow-up questions, and paraphrasing were used to stimulate more detailed responses, and to verify the interviewer’s initial interpretations. At the end of the interview, family members were asked if there was anything else they wanted the interviewer to know. The interviewer (first author) wrote field notes immediately after each interview. Audio recordings were uploaded to a secure platform and transcribed verbatim by the interviewer. Quotes presented in this paper are translated from Norwegian and edited for clarity.

### Analysis and interpretation

Interpretation of the material started during the interview, as the study’s aim and the interviewer’s horizon of understanding influenced what the researcher heard and the researcher’s interpretations of the participants’ answers. This interpretation influenced follow-up questions during the interviews. Similarly, transcriptions of interviews also involve an interpretation of the material (Brinkmann & Kvale, 2018). Hence, the study aims and preconceptions were crucial for the interpretations. Following these initial steps, the first author conducted the formal interpretation of the interviews by entering a dialogue with the texts. The researcher wrote memos with preliminary ideas and interpretations during this process. This dialogue was initiated by a careful reading of the interviews to gain an in-depth understanding of the fundamental meaning of the interviews, as the meaning of the whole text influences the understanding of the parts of the texts (Fleming et al., 2003). The first two interviews were read in their entirety by four members of the research group to ensure that all researchers had the same initial understanding of the fundamental meaning of the whole interview. The interpretation of the interviews continued with a reading where every sentence and section were investigated to expose its meaning, thus facilitating the first identification of themes and ideas. The sections were then related to the meaning of the whole text expanding this understanding. This process of analysing and interpretating continued in an iterative process between the texts, and the meaning in its entirety and sections of the material generated new interpretations that were subsequently applied to the entirety of the texts (Gadamer, 2013). Previous research and theoretical texts (the theoretical framework) were also included in this iterative process that expanded the understanding of the whole and the parts. When we felt that we had reached sufficient understanding, data passages and sections of the texts that seemed to be representative of the shared understandings were identified. These sections and passages were then translated to English by the first author. The write-up of the findings section and the discussion also facilitated new interpretations and new understanding of the subject matter.

### Ethical considerations

The principles of informed consent, confidentiality, and assessment of consequences for participants were observed throughout the entire study. All participants received written and oral information, were assured of confidentiality, and informed that they could withdraw from the study at any time. The Regional Ethics Committee (REK Case number 175,774) and Sikt—Norwegian Agency for Shared Services in Education and Research (Case number 248,550) permitted and supervised the study. Family members from Langerudhjemmet participated in an advisory user participation group. This advisory user participation group reviewed consent forms and information, interview guides, and interpretations.

### Trustworthiness and auditability

To ensure trustworthiness and auditability of the study we diligently detailed each stage of the research process while adhering to the Standards for Reporting Qualitative Research (SRQR) (O’Brien et al., 2014). Trustworthiness and interpretation validity were also reinforced as the researcher, who conducted the interviews, transcribed, analysed, and interpreted them (Brinkmann & Kvale, 2018). Final interpretations were reviewed by an advisory user participation group. Quotations from the interviews offer support for the interpretations presented in the findings.

## Findings

The interpretation of the interviews revealed that the family members were concerned that caregivers placed too much emphasis on resident autonomy when making care-related decisions. Caregivers prioritizing resident autonomy over resident wellbeing caused the family members to question their competence and worry for resident safety. From the perspective of the resident’s family, the resident’s wellbeing is the caregiver’s responsibility. The family members expressed that resident wellbeing should be the guiding principle for decision-making regarding resident care.

### Excessive focus on autonomy threatens resident wellbeing and safety

The family members expressed concern that nursing home staff placed too much emphasis on resident autonomy and would hence leave too many decisions, and the wrong kind of decisions, to the resident. One participant expressed.

(P3): I sometimes wonder that she is allowed to refuse. That she can say “no thanks” to food in the evening, and then have very low blood sugar in the morning. Then I think: “Where is the professional judgment?” Maybe it is because of goodwill? Because that’s how we usually relate to others?

The family members questioned the professional caregiver’s knowledge and competence when they entertained refusal too easily or left the wrong decisions to the resident. This led to worries that residents would not receive necessary care in the nursing homes. The family members attributed this excessive focus on residents’ choice to goodwill and misguided attempts to preserve resident autonomy, along with an overly fearful attitude towards coercion.

(P3): I think that when you’re a professional, you’re very afraid to be accused of being coercive.

From the perspective of the family members, a resident’s refusal did not always mean that they made a deliberate choice to not receive care, or to not perform care activities. Rather, the resident’s refusal was interpreted as a sign of other issues.

(P5): I think that if Dad refuses these things, there’s something wrong, something that he doesn’t quite have control over. Then you must try to talk him into it. Make him come to his senses.

Family members also considered that the resident could have a change in values and priorities regarding their standards for appearance and hygiene.

(P10): I realised that those things became a chore to my dad, and he didn’t do as much of it anymore. He has always been such an incredibly decent guy… He never walked across the lawn, he always walked on the created path …. But eventually, things like that weren’t as important anymore. I realise that’s part of getting old ….

While mentioning her father’s changed values and priorities, this participant also attributed his refusal of basic care activities to him being in pain, indicating that her father’s values may not have changed as much as the effort needed to maintain his standards and values.

(P10): I’m sure it would have been easier if he wasn’t in pain, as what he’s saying is that he can’t stand it because it hurt. So, the fact that he’s in pain is really what makes him refuse.

The family members mentioned several barriers that contributed to residents refusing care, which caregivers should recognize and overcome to ensure proper care. Family members mentioned lack of motivation and initiative, lack of energy, misunderstanding caregivers’ expectations, a fear of being unable to meet caregivers’ expectations, discomfort with receiving help from someone new, shame due to loss of independence, and pain as reasons for residents refusing care. These barriers were mentioned by family members of residents with and without reduced cognitive capacity. The family members were very clear that identifying and overcoming these reasons were the responsibilities of professional caregivers.

(P6): They must try to understand what makes her not want to ….

However, this did not mean that family members wanted caregivers to disregard the resident’s opinions.

(P9): They shouldn’t be deprived of their opinions, that’s not what I mean. But sometimes it’s kind of like…. I think staff often give up way too quickly or it might be that they are not allowed to push or coax residents …. It might be that residents should decide for themselves…. But I’ve had a mother in a nursing home before, and if she said “no” one day, they simply left her.

### Resident wellbeing is the caregiver’s responsibility

Family members expected the nursing home staff to assume responsibility for the resident’s fundamental physical needs, social needs, and hygiene standards, as this care was originally provided to the residents by the family members.

(P8): I think they should be able to do it. Because I did it when the homecare services couldn’t. Yes, you must do it. It’s also part of the education, how you’re going to “turn that tile”, so to speak, to make it happen.

Assuming responsibility meant that one was responsible for finding ways to deliver care and meet resident needs even when residents refused care, as refusal had consequences that the resident could not take responsibility for.

(P6): If someone wants to jump off a balcony, it’s obvious that you must intervene. If she doesn’t want to clean and take care of herself, that becomes a consequence for her life and health in the end but takes some time…. And dental health is also a concern. I doubt that she’ll brush her teeth by herself, and I think that’s important—the things that we opt out of occasionally that have consequences later.

Family members also expressed that caregiver responsibilities should include maintaining the resident’s dignity.

(P3): There aren’t any goals left to achieve. Hence, it is more important to make sure she looks well-groomed. That’s how we as relatives think. A well-groomed exterior allows others to approach you in a different way than when you are not well-groomed. I think that the fact that she’s still treated with dignity is important to Mom, and she must look proper for that.

Hence, family members expected caregivers to assume responsibility for the dignity and personhood of residents, and to ensure that the resident’s needs were met despite their refusal.

(P11): Not everyone understands that she really wants to participate, even though she says no, and that they must persuade her.

The idea that caregivers should take responsibility when residents refused care was also evident in the expectations of the nursing home caregiver’s competence and training. Family members expected professional caregivers to have psychological knowledge and training in counteracting refusal and making residents cooperate.

(P5): I assume that when you work in a nursing home and have a medical background, you are both used to and trained to handle such situations.

### Resident wellbeing as a guiding principle

The family members emphasized that the care provided in the nursing home should always be guided by the resident’s needs and wellbeing. This meant that they expected caregivers to have knowledge and awareness of each individual resident and their unique personality.

The family members did not perceive the provision of care despite refusal as negative or harmful if the motive for ignoring refusal was to safeguard the resident’s needs.

(P7): I basically don’t think of it as negative because there is no malice to it. I think that if you have the resident’s wellbeing in mind, you should try to find a way of implementing these things. I think the people who work here want to do what’s best for her, but it’s not easy as she doesn’t always see things the same way as they do.

Overruling a resident’s wishes because of concerns with institutional routines rather than the needs of the individual resident, or to alleviate the professional caregiver’s own needs were not perceived as legitimate. The son of a female resident disclosed very disturbing experiences that clarify the indignity that occurs when the resident’s wellbeing is not the guiding principle.

(P4): It was neat and clean and orderly, but …. It was all those small episodes … Once when I came, she wanted to go to the toilet, but was told that she could just do it in her panties, because it was mealtime. You could forget about getting help to go to the bathroom at mealtime. Then she laid there with her trousers full, and the same person came back and asked her what she wanted for dinner! I mean, you can’t do that!

Although the family members stressed that it was the caregivers’ responsibility that residents were well groomed, and that refusal of care should be overruled, they did not want residents to feel coerced or disrespected. The family members stressed that caregivers should find ways to overcome refusal and deliver care in ways that provide residents with an experience as good as possible.

(P6): I think they should find a way to care for her, but at the same time, you shouldn’t drag her with physical force into the shower. There’s a middle ground, where you must try to find the ways to make it happen. The ends do not justify the means. However, it depends on how important it is.

Residents’ expressions of wellbeing and contentment after the completion of a care activity were perceived as crucial for settling situations where professional caregivers were unsure if refusal should be respected. If residents expressed positive feelings of wellbeing after the care activity, the family members perceived that caregivers should continue to deliver care despite the resident’s refusal. If a resident expressed resentment after completion, the resident’s refusal should be respected henceforth.

## Interpretive discussion

The family members were concerned that caregivers overemphasized the resident’s right to choose and that they were too fearful of coaxing residents, thus leaving too many decisions and the wrong kind of decisions to the resident. Previous studies have indicated that residents’ family members in nursing homes can act both as a support and obstruction to resident autonomy (Moilanen, Suhonen, et al., 2021). The family members participating in this study expressed that an overemphasis on autonomy can compromise quality care and pose a risk to the resident’s health, safety, and dignity. Conversely, caregivers have been found to view the respect for residents’ wishes and advocacy on their behalf as a fundamental aspect of quality care (Moilanen, Suhonen, et al., 2021). This indicates a disagreement regarding the significance of autonomy for good care in nursing homes.

The family members expressed that residents should not be allowed to choose whether to receive/perform care activities in all situations. The consequences of some situations, although not immediately life- or health-threatening, may be too serious that residents should not be allowed to choose freely. This could, for instance, be when residents refuse to eat, are reluctant to brush their teeth, or neglect to maintain their standards of appearance. This can be interpreted as family members advocating for caregivers to prioritize the wellbeing (beneficence) of residents over their autonomy in certain situations, while caregivers in the nursing home prioritize resident autonomy (Moilanen, Suhonen, et al., 2021). Historically, nursing homes have transitioned from a paternalistic approach, to prioritizing residents’ autonomy. Does this mean that the family members feel that this shift has gone too far? Or is this an indication that the family members do not fully appreciate the importance of autonomy in preserving the dignity of the residents?

Notably, family members said that the residents’ refusal often did not reflect the residents’ true values and goals. This suggests that family members consider the residents’ previous values and standards as the accurate representation of their personhood. Family members have previously been found important in aiding residents’ continuity of values, beliefs, and personal identity, which has been associated with adjustment, satisfactions, and healthy transition to the nursing home (Fitzpatrick & Tzouvara, 2019). Conversely, caregivers might view the choices made by residents and their expressed values as the genuine reflection of the residents’ wishes and personhood. During the interviews, one family member (P10) expressed that her father’s values and standards had changed as he grew older, that her father who had always been very proper started to neglect the standards and values he had previously held. She was expressing the idea that a reduction of standards and values was a “part of getting old”. This idea that values and standards for appearance are lowered with elderly residents has also been described by caregivers working with residents suffering dementia (Mortensen et al., 2022). Caregivers in nursing homes verbalized that nursing home residents did not have the same standards for hygiene and appearance anymore. This expectation of lowered standards in the elderly was interpreted as a reaction to the cognitive dissonance caregivers felt when residents refused care (Mortensen et al., 2022). This suggests that there may be underlying assumptions that elderly individuals refuse help with hygiene and appearance because of alterations in their values and standards. However, from the family’s perspective, this perceived reduction in compliance to previous standards did not solely reflect changes in the residents values and standards. The participating family members also expressed that the refusal of care was caused by barriers to the execution of the necessary actions. These barriers made it much more difficult for the residents to uphold their previous standards. If we interpret the refusal as a consequence of these barriers and not as an indication of the residents’ values, then this would mean that the refusal was not truly autonomous because an autonomous choice reflects the person’s second-order values and desires, as opposed to immediate desires (Killmister, 2018).

Caregivers who are unaware of the resident’s life, may find it challenging to ascertain if a resident’s refusal is in accordance with their true values and standards or caused by such barriers. For example, the daughter (P10) who first described how her father’s standards had changed, later in the interview expressed that her father being in pain was the real reason behind his refusal. Cultural and linguistic differences between residents and caregivers can make distinguishing the resident’s true values and goals even more challenging for caregivers (Xiao et al., 2022). Meanwhile, family members share the resident’s cultural values and language, and remember who the resident used to be. This increases continuity with the resident’s previous life, values, goals, and personhood (Fitzpatrick & Tzouvara, 2019), and strengthens family members’ ability to recognize when the resident’s refusal is not in line with their typical behaviour. This discrepancy between the resident’s present behaviour and choices and their previous behaviour and values also seemed to trigger their family members to search for explanations other than such behaviours being expressions of the residents’ will. As one participant (P5) explained, “*If Dad refuses these things, there’s something wrong”*. Interestingly the family members also expressed that residents’ expressions of wellbeing and contentment after completion of the care activities were crucial in situations where caregivers were unsure if refusal of care was an autonomous choice or a challenge to overcome. If residents showed signs of anger or feelings of manipulation after the care activity, their initial refusal was to be considered a reflection of their true wishes and values. Hence, the family members seemed to understand reactions post-care as better indicators of residents’ true values and standards than their initial refusal.

The family members expected the caregivers to take responsibility for the resident’s wellbeing. This responsibility involved basic care, taking charge of resident’s social needs through participation in activities, and ensuring that the resident’s hygiene and appearance was up to their usual standards. The family members perceived this as crucial for maintaining the resident’s dignity. Notably, a family member (P3) said that the residents did not have any goals left to achieve anymore. Independence and the pursuit of goals are central norms that constitute personal and social dignity (Caspari et al., 2018; Killmister, 2020). Similarly, maintaining norms of appearance contributes to dignity (Killmister, 2020). When we consider that family members believe that residents do not have (P3) *“goals left to achieve”*, the dignity of appearance, *“looking well-groomed”*, may be the most important source of dignity left, ensuring that the resident still feels respected and is treated with dignity by others. This differs from previous research on dignity and autonomy, which emphasizes the need to support resident autonomy and freedom of choice to respect their dignity (Caspari et al., 2018; Davison et al., 2019; Jacobson & Silva, 2010; Moilanen, Suhonen, et al., 2021). This would explain why caregivers taking responsibility not only for residents’ health and safety but also for residents being well-groomed and residents’ participation in activities was so important to the family members.

Although the family members expressed that there was too much focus on autonomy, they also did not want caregivers to force or coerce residents into receiving care. They expected caregivers to find ways to overcome the resident’s refusal without disrespecting residents’ opinions or using coercion. To ensure that residents are not disrespected, the family members suggested that caregivers use resident wellbeing as a guiding principle to decide when and how they influence residents’ decisions and actions. First, the motive and reason for influencing residents’ decisions and actions should be to safeguard resident wellbeing—their needs, personhood, and dignity. Other motives for overruling resident choices were considered illegitimate. This corresponds with paternalism as disregarding someone’s desires for their own good (Killmister, 2018). Second, the family members expected caregivers to always regard the resident’s feelings both during the care activity and after the care was delivered. This expectation aligns with previous research emphasizing that caregivers should consider a resident’s feelings during care by choosing less invasive methods and being mindful of how these methods are applied (Lohne et al., 2017). This aligns with the concept of soft paternalism (Killmister, 2018). Hence, it seems that family members support the use of soft paternalism in the care of their residents.

### Strengths and limitations

This study was conducted during the COVID-19 pandemic, with the limitations that the pandemic entailed. Family members were not allowed to visit residents as often as before and after the pandemic. When family members visited residents, they were instructed to move to the resident’s room and stay there during the visit. This may have restricted family members’ insight into the interactions between residents and staff, and decision-making in nursing homes during this period. The COVID-19 restrictions also influenced the family members to be highly motivated for participation in the interviews as they considered the interviews as a method of gaining access and influence during COVID-19 restrictions.

Another possible limitation of the study is that the participants were recruited by registered nurses who were responsible for the participating units. This might have biased the sample, as the responsible nurse may have recruited participants who were cooperative and portrayed the nursing home in a favourable light. To counter this limitation, participant recruitment was conducted following an observation period. During the observation period, the researcher and the responsible registered nurse reached a consensus on the inclusion criteria and the importance of a diverse sample. We now believe that recruitment by the responsible nurse was a strength of the study. As the nurse was involved in daily care and had extensive knowledge of both the residents and their family members, this study was able to achieve a varied sample. We also consider the co-authors’ extensive experience with qualitative interviews and hermeneutic interpretation as a strength of the study, as the study’s underlying preconceptions and its influence on the findings (Gadamer, 2013) was discussed in detail throughout the process.

A stage of Flemming’s Gadamerian-based research method (Fleming et al., 2003) involves gaining understanding through dialogue with participants. This stage involves the researcher and participant working together to achieve a fusion of perspectives. As understanding depends on the historical situation, Fleming et al. (2003) recommends that the interviewer speak to the participant two or three times. Repeated interviews were not achievable in this study. To address this limitation, the interviews were conducted after a period of participant observations in the nursing home, which gave the participants and the researcher common frame of reference before the interview. The researcher validated interpretations during the interviews by asking follow-up questions and paraphrasing interpretations, and final interpretations were discussed with the user participation group.

## Conclusion

Our findings indicate a disagreement between the family members and the nursing home caregivers regarding the significance of upholding resident autonomy to respect residents’ dignity. The family members held that not all instances where residents refused care reflect autonomy situations as the care refusal often does not reflect the resident’s true values and standards. Rather, the family members observed that often, care refusal stems from barriers that render necessary care actions difficult. In situations where residents refuse essential care or when the refusal does not align with the residents second-order values, the family members suggested that caregivers should strive to understand the causes of refusal and seek non-coercive ways to navigate it. Hence, the participating family members seemed to endorse the use of soft paternalism in nursing homes to safeguard residents’ wellbeing and dignity. Our findings suggest that when residents resist care, caregivers should not only assess the residents’ capacity for autonomy but also identify potential barriers to care activities and evaluate if the conditions for autonomy are fulfilled. Further research is needed to examine the validity of this new perspective in both local and broader contexts.

## Supplementary Material

SRQR_Checklist Family caregivers perspectives_clean.docx

Autor Biography.docx

## Reporting method

This study adheres to EQUATOR guidelines for qualitative research using the SRQR Standards for Reporting Qualitative Research (O’Brien et al., 2014).
