# Supplementary material for: A qualitative study of family members’ perspectives regarding decision-making for nursing home residents’ care
Source: Int J Qual Stud Health Well-being. 2024 Jun 21;19(1):2370545. doi: 10.1080/17482631.2024.2370545 (PMC11195484; doi:10.1080/17482631.2024.2370545)
Supplement: Autor Biography.docx [file ZQHW_A_2370545_SM1264.docx]

# A short biographical note for each author:

**Anne Helene Mortensen** has been a dedicated nurse since 1996 and is currently a University Lecturer at the Institute for Nursing and Health Promotion at OsloMet. She holds a Cand San degree from the University of Oslo. Mortensen is part of the Dignity and Ethics research group at OsloMet and is currently pursuing her PhD. Her research is focused on investigating stakeholder experiences with soft paternalism and nudging in nursing homes, using a hermeneutic interpretation approach.

**Dagfinn Nåden** is an esteemed Professor Emeritus at Oslo Metropolitan University (OsloMet). He remains an active member of the Dignity and Ethics research group, which he formerly headed. Nåden completed his doctoral studies at Åbo Akademi University under prof. Katie Eriksson’s leadership, focusing on the art dimension of nursing.

Despite his emeritus status, he continues to engage in active research, particularly in the fields of Gadamer's Hermeneutic interpretation, ethics and dignity research.

In addition to his contributions at OsloMet, Nåden has held prestigious positions in academic institutions across Europe. He serves as a Docent at Åbo Akademi University in Wasa, Finland, and as a Visiting Professor at the University of Surrey in Guildford, UK. His international experience and ongoing research make him a respected figure in his field.

**Dag Karterud** has worked as a clinical nurse caring for children with epilepsy, headed a department for elderly people, and provided nursing services in various healthcare facilities for drug abuse, as well as in a rehabilitation center. He completed his Master’s studies at the University of Oslo, and his PhD at Åbo Academy in Vasa, Finland, focusing on Caring Science. His thesis examined how to ensure ethics in healthcare when patients face existential issues due to severe illness. Dag has been an educator and researcher at OsloMet for many years, primarily in the Bachelor's program, but has also supervised Master's and PhD students. He has been a fellow researcher in Assisted Living, a multiprofessional research and innovation project studying how to implement technology in healthcare. He has also served as the head of studies and head of a department at the Faculty of Health Sciences.

**Ann Gallagher** is an experienced professional and academic with a wealth of experience in health and social care, education, research, and editing. Ann is a registered nurse (adult and mental health) and has a PhD in Professional Ethics. She has worked mainly in London and South East England. She is Head of the Department of Health Sciences at Brunel University London. Ann is a Fulbright Scholar, a Fellow of the Royal College of Nursing and American Academy of Nursing and serves as the Editor-in-Chief of the journal 'Nursing Ethics'.

**Vibeke Lohne** is a respected Professor in Nursing Science at the Department of Acute and Critical Care at Oslo Metropolitan University (OsloMet). She earned both her Master's degree and PhD (Dr.polit) in Nursing Science from the University of Oslo, solidifying her expertise in the field. Dr. Lohne leads the Dignity and Ethics research group at OsloMet. Her research primarily centers on the themes of hope and dignity, with a particular focus on its implications for patients, family caregivers, and healthcare personnel. Throughout her academic career, Dr. Lohne has consistently aimed to enhance understanding in these areas, contributing valuable insights that help shape patient care and healthcare practices.
